# The Hospital Library and Charities Bureau

**Published:** 1905-03-25

**Authors:** 


### THE HOSPITAL LIBRARY AND CHARITIES BUREAU.

This institution has been founded as a centre of reference in all matters concerning the establishment and management of charities. It is open to all interested in the subject on payment of a small annual fee, and inquiries by members can be made by post. Write for prospectus to the Librarian, The Hospital Buildings, 28 & 29 Southampton Street, Strand.

Inquiries of the librarian are answered in this column free of charge. Inquiries to be answered promptly by post must be accompanied by a fee of 2s. 6d.

The Librarian announces the addition of 24 volumes of the Encyclopædia to the Library for the use of members.

I am very anxious to get a tennis court for the nurses made in the spare ground round this hospital, and should be greatly obliged for any information as to cost, best material, etc.—*Inquirer.*

We have consulted an expert on this query, and have received the following reply:—With regard to the tennis court, I should say from my own experience that the cheapest and most satisfactory material is ashes. We laid down one at the Branch Seaman's Hospital a year or two ago, and it answers admirably. About three-quarters of an inch of ashes are put on once a year and well rolled in, and the court is watered and rolled in the summer every day, the boy who is employed in the laboratories finding time to do this part of the work. The ashes cost nothing, as they are the waste product of the steam boiler. I don't think that asphalt will be found satisfactory. It is pretty certain to crack, unless it is done with Seyssel asphalt in the most expensive way possible. I have heard of a mixture of ashes and tar being used and proving a good material, but I have no personal experience of this.

